# Patientenzahlen im Rahmen der COVID-19-Pandemie in einer zentralen Notaufnahme

**DOI:** 10.1007/s10049-020-00757-w

**Published:** 2020-07-08

**Authors:** T. Tschaikowsky, A. Becker von Rose, S. Consalvo, P. Pflüger, P. Barthel, C. D. Spinner, B. Knier, K.-G. Kanz, M. Dommasch

**Affiliations:** 1 Fakultät für Medizin, Klinikum rechts der Isar, Innere Medizin I, Technische Universität München, München, Deutschland; 2 Zentrale interdisziplinäre Notaufnahme, Klinikum rechts der Isar, Technische Universität München, Ismaninger Str. 22, 81675 München, Deutschland; 3 Fakultät für Medizin, Klinikum rechts der Isar, Innere Medizin III, Technische Universität München, München, Deutschland; 4 Fakultät für Medizin, Klinikum rechts der Isar, Unfallchirurgie, Technische Universität München, München, Deutschland; 5 Fakultät für Medizin, Klinikum rechts der Isar, Innere Medizin II, Technische Universität München, München, Deutschland; 6 Fakultät für Medizin, Klinikum rechts der Isar, Neurologie, Technische Universität München, München, Deutschland

**Keywords:** Notfall, Patientenaufkommen, Coronavirus, SARS-CoV‑2, Kollateralschaden, Emergency, Patient numbers, Coronavirus, SARS-CoV‑2, Collateral damage

## Abstract

**Hintergrund:**

Seit Ende März wurde deutschlandweit das Gesundheitswesen auf einen Notbetrieb umgestellt, um Ressourcen für die sich ausbreitende Coronavirus-disease-2019-Pandemie (COVID-19-Pandemie) zu schaffen. Ziel der Arbeit ist es, das Aufkommen von Notfallpatienten zur Zeit der Pandemie zu untersuchen, um Rückschlüsse auf den Einfluss der COVID-19-Pandemie auf das Patientenaufkommen in einer Notaufnahme ziehen zu können.

**Material und Methoden:**

Im Rahmen einer deskriptiven epidemiologischen Studie wurden in dem Zeitraum vom 01.02. bis zum 30.04.2019 sowie vom 01.02. bis zum 30.04.2020 patientenbezogene Daten von insgesamt 19.357 Fällen in der zentralen Notaufnahme des Klinikums rechts der Isar erhoben und anonymisiert ausgewertet.

**Ergebnisse:**

Trotz steigender Patientenzahlen von 2019 auf 2020 kam es von Februar auf März 2020 zu einem deutlichen Abfall der Notfälle bis auf ein Niveau unter das von 2019, der im April weiter anhielt. Dies betraf insbesondere den Fachbereich Unfallchirurgie mit einem Rückgang des mittleren Patientenaufkommens um etwa 40 %.

Im Hinblick auf die Beschwerdebilder im März 2020 zeigte sich, dass ein vermehrtes Aufkommen von Unwohlsein (+47 %) und Atemproblemen (+36 %) zu verzeichnen war, wohingegen Rückenschmerzen (−41 %), Wunden (−29 %), thorakale (−24 %) sowie abdominelle Schmerzen (−23 %) deutlich seltener vertreten waren als im Vorjahr. Bezogen auf den Schweregrad der Beschwerden wirkte sich der Rückgang vor allem auf Beschwerdebilder mit niedriger Dringlichkeitsstufe aus.

**Schlussfolgerung:**

Im Rahmen der COVID-19-Pandemie kam es zu einem deutlichen Rückgang des Patientenaufkommens in einer der größten Notaufnahmen in München. Dies sollte bei bestehenden Krankenhauskapazitäten vermieden werden, um potenziell gesundheitlichen Schäden durch eine aufgeschobene oder ausbleibende notfallmäßige Vorstellung vorzubeugen.

## Abstract (en)

**Background:**

Since end of March, the health care system in Germany has been placed into a state of emergency in order to gain resources for the spreading coronavirus disease 2019 (COVID-19) pandemic. The overall goal of this study is to evaluate the number of emergency room patients at the time of the pandemic in order to draw conclusions about the influence of the COVID 19 pandemic on the number of patients in an emergency department.

**Materials and methods:**

With this descriptive epidemiologic study we collected and analyzed anonymized patient-related data of 19,357 cases presenting to the emergency department of the Klinikum rechts der Isar (Munich) from 01 February 2019 to 30 April 2019 and from 01 February 2020 to 30 April 2020.

**Results:**

Despite an increase in the number of patients from 2019 to 2020, there was a significant drop in the number of emergencies from February to March 2020 and proceeding in April to a level below that of 2019. This was particularly observed in the field of trauma surgery, with a 40% decrease in the number of patients. With regard to the individual complaint patterns in March 2020, it was found that an increased incidence of malaise (+47%) and breathing problems (+36%) was recorded, whereas back pain (−41%), wounds (−29%), thoracic (−24%) and abdominal pain (−23%) were significantly less common than in the previous year. In terms of the severity of the complaints, the decline was mainly due to complaints with a low degree of urgency.

**Conclusion:**

In the course of the COVID-19 pandemic we observed a significant decline in the number of patients in one of the largest emergency rooms in Munich. This has to be avoided with existing hospital capacities, in order to prevent potential damage to health caused by postponed or missing emergency presentations.

## Einleitung

Aufgrund der steigenden Infektionen mit der neuartigen Atemwegserkrankung Coronavirus-disease-2019 (COVID-19) in China hat die Weltgesundheitsorganisation (WHO) am 30.01.2020 eine „gesundheitliche Notlage von internationaler Tragweite“ ausgerufen [17]. Gegen Ende Februar stiegen die Fallzahlen auch in Europa. Insbesondere in Norditalien kam es zu einem sprunghaften Anstieg an COVID-19-Patienten und auch in Deutschland gab es erneut Fälle von Infektionen mit SARS-CoV‑2, nachdem es bereits im Januar nur einen Eintrag in der Region München gab [3].

Am 11.03.2020 wurde vonseiten der Weltgesundheitsorganisation der COVID-19-Ausbruch offiziell zu einer Pandemie erklärt [16]. Die nationale Seuchenschutzbehörde, das Robert Koch-Institut, bewertete die Gefährdung für die Gesundheit der Bevölkerung in Deutschland am 17.03.2020 insgesamt als „hoch“ [7]. Analog hierzu hat die Bayerische Staatsregierung am 16.03.2020 den Katastrophenfall ausgerufen und Ausgangsbeschränkungen erlassen, die ab dem 21.03.2020 bis zum 05.05.2020 galten und am 06.05.2020 durch Kontaktbeschränkungen ersetzt wurden [1, 4, 6, 11]. Im Zusammenhang mit dem Katastrophenfall haben das Bayerische Staatsministerium des Innern, für Sport und Integration sowie das Bayerischen Staatsministeriums für Gesundheit und Pflege am 24.03.2020 eine Allgemeinverfügung erlassen, in der unter anderem die Organisation der Krankenhausbelegung, Neukonzeption der IT-Steuerung und Meldepflichten, Schaffung zusätzlicher Kapazitäten, COVID-19-Koordinierungsgruppen der Krankenhäuser sowie organisatorische Maßnahmen für Krankenhäuser angeordnet wurden [5].

Entsprechend der vorhandenen Pandemie- und Notfallpläne wurden im Raum München schon nach dem Auftreten der ersten COVID-19-Fälle Vorkehrungen im Gesundheitswesen getroffen. Auch aufgrund der Berichterstattung aus Italien, Frankreich und Spanien wurden Krankenhauskapazitäten umfunktioniert und Intensivbetten geschaffen, um einem möglicherweise bevorstehenden großen Ansturm an Patienten gerecht zu werden [2].

Im Rahmen dieser Vorhaltemaßnahmen wurden Betten in Fachbereichen, die hauptsächlich elektive Medizin betreiben, reduziert, um andere Bereich, wie z. B. die Notaufnahmen, zu stärken. Mit dem Sonderlagenmodul IVENA (mainis IT-Service GmbH, Offenbach am Main, Deutschland) melden die Kliniken mehrmals täglich den Ist-Stand der Betten an die Sicherheits- und Gesundheitsbehörden.

Die COVID-19-Fälle stiegen täglich weiter an, aber aufgrund der deutlichen und strikten Ausgangsbeschränkungen verlängerte sich auch die Verdopplungszeit der Viruserkrankungen, sodass es erfreulicherweise zu keiner Überlastung der Krankenhäuser in Deutschland gekommen ist.

Alle Vorhaltemaßnahmen, die in den Monaten getroffen wurden, hatten zum Nebeneffekt, dass die medizinische Versorgung auf einen Notbetrieb umgestellt wurde. Die hierdurch entstandenen gesundheitlichen und volkswirtschaftlichen Schäden sind noch nicht absehbar. Diese Arbeit soll das Patientenaufkommen zu Beginn und nach Abflachen der wohl ersten COVID-19-Welle in Deutschland aufzeigen und kann für weitere Pandemieplanungen im Bereich der Notaufnahmen miteinbezogen werden.

## Methoden

### Zentrale Notaufnahme des Klinikums rechts der Isar im Kontext

Das Klinikum rechts der Isar (RDI) der Technischen Universität München weist gemäß § 136c Absatz 4 des 5. Buches, Sozialgesetzbuch (SGB V) die Voraussetzungen für eine Klinik der „umfassenden Notfallversorgung – Stufe 3“ auf [9]. Die zentrale Notaufnahme (ZNA) des Klinikums gehört zu den größten im Rettungsdienstbereich München. Jährlich werden mehr als 40.000 Notfälle in der ZNA behandelt. Sie ist für jegliche Notfälle ausgerüstet und verfügt über einen Schockraumbereich mit Computertomographie, für den ein interdisziplinäres Team aus den Fachbereichen Chirurgie, innere Medizin, Neurologie, Anästhesie und Radiologie vorgehalten wird. Hauptsächlich werden in der ZNA Patienten aus den Fachbereichen Chirurgie, innere Medizin sowie Neurologie behandelt. Zusätzlich stehen am Klinikum Spezialambulanzen der Bereiche Urologie, Gynäkologie, Mund-Kiefer-Gesichtschirurgie, Hals-Nasen-Ohren-Heilkunde und Augenheilkunde zur Verfügung.

Im Zuge der SARS-CoV-2-Pandemie erfuhr die ZNA eine grundlegende Umstrukturierung in einen räumlich getrennten infektiösen und nichtinfektiösen Bereich. Die Patienten werden analog eines an die Gegebenheiten des Klinikums angepassten COVID-19-Algorithmus anhand ihrer Symptome und Temperatur in die jeweiligen Bereiche getrennt [15, 18].

### Manchester Triage System

Bei Vorstellung der Patienten in der ZNA erfolgt nach personeller Identifikation eine medizinische Ersteinschätzung, auch „Triage“ genannt, gemäß einem validierten System, dem Manchester Triage System (MTS; [10]). Anhand von insgesamt 50 Beschwerdebildern und entsprechenden Präsentationsdiagrammen wird die Dringlichkeit des Notfalls festgestellt. Hierdurch wird die maximale Zeit bis zum ersten Arztkontakt definiert und nach einem Ampelprinzip von „blau“ für nichtdringend bis „rot“ für akute Lebensgefahr eingeteilt (Tab. 1).

**Table 1:** 

Triagefarbe	Dringlichkeit	Zeitpunkt ärztlicher Kontakt	Beschwerden
*Blau*	Nichtdringend	<120 min	Seit Längerem bestehende Beschwerden
*Grün*	Normal	<90 min	Subakute bis akute Beschwerden ohne Risikozeichen
*Gelb*	Dringend	<30 min	Akute Beschwerden mit Risikozeichen, z. B. Blutungszeichen, mäßige Schmerzen, offene Frakturen, Fehlstellungen, neurologisches Defizit oder niedrige periphere Sauerstoffsättigung (<95 % unter Raumluft)
*Orange*	Sehr dringend	<10 min	Starke akute Beschwerden, z. B. akute Atemnot, Bluterbrechen, stärkste Schmerzen, insbesondere Thoraxschmerz, veränderter Bewusstseinszustand oder sehr niedrige periphere Sauerstoffsättigung (<90 % unter Raumluft)
*Rot*	Sofort	0 min	Akute Lebensgefahr, z. B. bei akutem Schockgeschehen, gefährdetem Atemweg, lebensbedrohlicher Blutung, anhaltendem Krampfanfall oder Hypoglykämie

### Fachbereiche

Die verschiedenen Fachdisziplinen wurden hinsichtlich einer besseren Übersicht in übergeordnete Fachbereiche zusammengefasst. „Chirurgie“ beinhaltet alle chirurgischen Subdisziplinen wie Unfall‑, Viszeral‑, Thorax‑, Gefäß‑, Mund-Kiefer-Gesichts‑, Neurochirurgie und plastische Chirurgie. Die Fachbereiche innere Medizin und Neurologie werden im Folgenden als „Konservativ“ zusammengefasst. Aufgrund der geringen Fallzahlen werden zur besseren Übersicht alle anderen Fachbereiche, wie Psychiatrie, Urologie, Pädiatrie, Gynäkologie und Geburtshilfe, im Folgenden als „Andere“ aufgeführt.

### ERPath

In der ZNA des Klinikums RDI werden alle patientenbezogenen Daten mittels eines Notaufnahmeninformationssystem (ERPath, eHealth-Tec Innovations GmbH, Berlin, Deutschland) erhoben und gespeichert [8]. Das Produkt ermöglicht eine vereinfachte und an die Notaufnahme angepasste Prozessteuerung inklusive Ersteinschätzung und Dokumentation. Es werden alle personenbezogenen Daten erhoben und gesichert. Das Triageergebnis wird mit Beschwerdebild, dessen Dringlichkeit und genauem Zeitstempel dokumentiert. Dies gilt auch für alle erhobenen Vitalparameter, verabreichte Medikamente und Therapien. Zudem werden alle administrativen Prozesse und klinische Untersuchungsbefunde, wie Anamnese, körperliche Untersuchung, Diagnostik und Konsile, systematisch erfasst.

### Datenerhebung

Im Rahmen einer deskriptiven epidemiologischen Studie wurden in dem Zeitraum vom 01.02.2019 bis zum 30.04.2019 sowie 01.02.2020 bis 30.04.2020 die in ERPath erhobenen Daten ausgewertet. Die Daten der im Beobachtungszeitraum erhobenen Notfälle wurden anonymisiert aus dem IT-System ERPath extrahiert und ausgewertet. Eine Signifikanz wird durch *p*-Werte <0,05 angezeigt. Es wurde keine Korrektur für mehrfache Tests vorgenommen. Für das Alter wurde der Mann-Whitney-U-Test, für alle anderen Faktoren der χ^2^-Test angewendet (SPSS Statistics for Windows, Version 25.0., IBM, Armonk, NY, USA; Microsoft Excel 2019; Microsoft Office, Redmond, WA, USA).

## Ergebnisse

### Basischarakteristika

In Tab. 2 sind die Baseline-Charakteristika der Patienten im Beobachtungszeitraum Februar bis März für die Jahre 2019 und 2020 aufgeführt. Das Alter der Patienten lag im Mittel bei 48 ± 21 bzw. 50 ± 21 Jahre. Der Anteil an männlichen Patienten lag bei 52 % respektive 53 %. Der Großteil der Notfallpatienten stellte sich selbstständig vor. Die Stufen grün und gelb waren in beiden Jahren erwartungsgemäß die am häufigsten im Rahmen der Triage gewählten Dringlichkeiten.

**Table 2:** 

Charakteristika		2019 (*N* = 9795)	2020 (*N* = 9562)	*p*-Wert
*Alter, MW* *±* *SD*		48 ± 21	50 ± 21	<0,001
*Geschlecht, n (%)*	Männlich	5085 (52 %)	5108 (53 %)	0,036
*Einweisungsart, n (%)*	Selbst	7118 (73 %)	6144 (64 %)	<0,001
	Rettungsdienst	1837 (19 %)	2732 (29 %)	<0,001
	Keine Daten	840 (9 %)	686 (7 %)	<0,001
*Dringlichkeit, n (%)*	Blau	1164 (12 %)	821 (9 %)	<0,001
	Grün	5426 (55 %)	4618 (48 %)	<0,001
	Gelb	2560 (26 %)	3487 (37 %)	<0,001
	Orange	486 (5 %)	519 (5 %)	0,144
	Rot	159 (2 %)	117 (1 %)	0,019
*Fachbereich, n (%)*	Chirurgie	6391 (65 %)	5051 (53 %)	<0,001
	Konservativ	3326 (34 %)	4345 (45 %)	<0,001
	Andere	78 (1 %)	166 (2 %)	<0,001
*Entlassart, n (%)*	Ambulant	7816 (80 %)	7159 (75 %)	<0,001
	Stationär	1976 (20 %)	2395 (25 %)	<0,001
	Verstorben	3 (0 %)	8 (0,1 %)	0,122

Bezüglich der Fachbereiche in den Jahren 2019 und 2020 hatte Chirurgie mit 65 % bzw. 53 % den größten Anteil, gefolgt von Konservativ mit 34 % bzw. 45 % und Andere mit 1 % bzw. 2 %.

### Patientenzahlen gesamt

Im Februar 2019 wurden in der zentralen Notaufnahme (ZNA) insgesamt 2941 Patienten behandelt. Im Mittel entspricht dies in etwa 105 Patientenkontakten täglich. Im Februar 2020 wurden insgesamt 3796 (davon 0 COVID-19-)Patienten versorgt, das entspricht also durchschnittlich etwa 131 Patientenkontakte pro Tag. Somit zeigt sich im Jahr 2020 im Vergleich zum Vorjahr ein Mehraufkommen von 26 %.

Im März 2019 wurden insgesamt 3333 Patienten in der ZNA behandelt (108 Patienten pro Tag). Im März des Folgejahrs waren es 3101 (davon 97 COVID-19-)Patienten (100 Patienten pro Tag). Demnach kam es im März 2020 im Vergleich zum Vorjahr sogar zu einem Abfall der Notfallpatienten um etwa 7 %.

Im April 2019 wurden in der ZNA insgesamt 3521 Patienten behandelt (117 Patienten pro Tag). Im April des Jahrs 2020 beliefen sich die Zahlen auf 2665 (davon 56 COVID-19-)Patienten (89 Patienten pro Tag), was einem Rückgang an Notfallpatienten von etwa 24 % entspricht.

**Figure 1:**
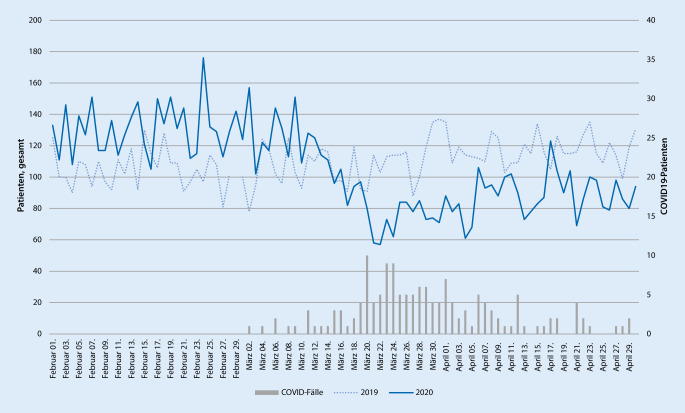

Die Patientenzahlen im untersuchten Zeitraum 2019 waren weitgehend konstant mit einer Abweichung von etwa 3 Patienten pro Tag und zeigten von Februar bis April einen Anstieg von etwa 12 Patienten pro Tag. Im Jahr 2020 dagegen zeigt sich von Februar bis April ein deutlicher Abfall der Patienten um etwa 42 Patienten pro Tag, was einem Rückgang von etwa 32 % pro Tag entspricht (Abb. 1).

### Fachbereiche

Im Fachbereich „Konservativ“ wurden im Zeitraum Februar bis April 2019 insgesamt 3326 Patienten behandelt, im selben Zeitraum 2020 waren es insgesamt 4345 Patienten, was einem signifikanten Mehraufkommen von 31 % entspricht (*p* < 0,001).

Im Vergleich der Monate Februar, März und April im Jahr 2019 waren die Patientenzahlen weitestgehend konstant und es zeigte sich eine leichte Schwankung von maximal etwa 3 Patienten pro Tag. Demgegenüber kam es im Jahr 2020 zu einem deutlichen Abfall von Februar zu März und weiter zu April, nämlich um etwa 11 bzw. 15 Patienten pro Tag, was einem relativen Rückgang von 27 % entspricht.

**Figure 2:**
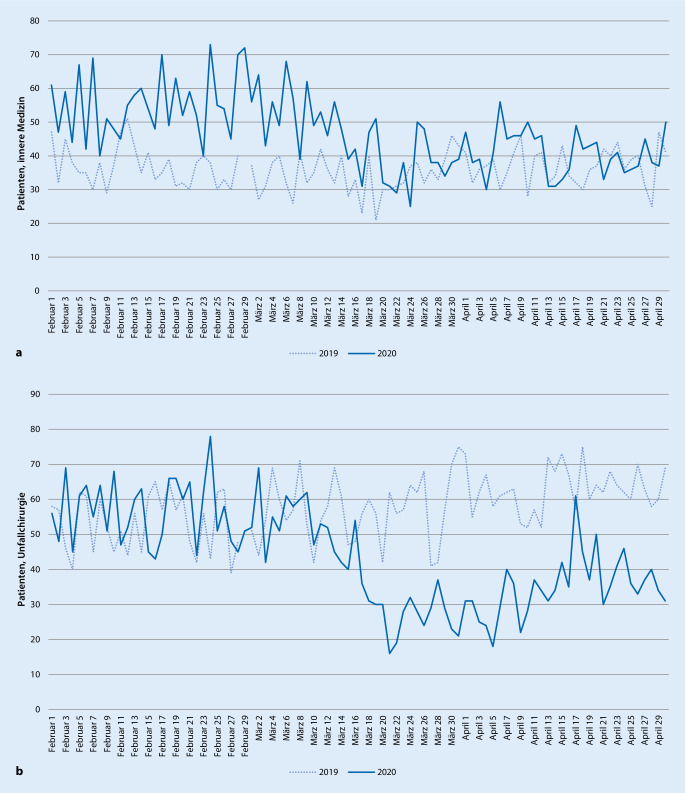

Dies betrifft insbesondere den untergeordneten Fachbereich „innere Medizin“. Während das Patientenaufkommen im Bereich „innere Medizin“ insgesamt gesehen im Verlauf der Jahre also stieg, fielen die Patientenzahlen im Jahr 2020 von Februar bis April deutlich ab, während sie im Vorjahr weitgehend konstant blieben (Abb. 2a).

Im Fachbereich „Chirurgie“ wurden im untersuchten Zeitraum 2019 insgesamt 6391 Patienten behandelt, wohingegen im selben Zeitraum 2020 insgesamt 5051, also signifikant weniger Patienten gesehen wurden (*p* < 0,0001). Von Februar auf März bis April desselben Jahrs zeigte sich für 2019 ein Anstieg der Patientenzahlen um etwa 6 bzw. 7 Patienten pro Tag. Im Jahr 2020 fielen die täglichen Patientenkontakte von Februar auf März und bis April stark ab, um etwa 20 bzw. 27 Patienten pro Tag.

In der Subdisziplin Unfallchirurgie wurden im Beobachtungszeitraum 2019 insgesamt 5136 Patienten versorgt. Im gleichen Zeitraum des Jahrs 2020 waren es 3944 Patienten. Dies entspricht einem Abfall des Patientenaufkommens um etwa 23 % bzw. 14 Patienten pro Tag. Umso deutlicher wird dies, wenn man die Änderung der Patientenzahlen im Monatsvergleich betrachtet (Abb. 2b). Hier zeigt sich für das Jahr 2019 ein leichter Anstieg des Patientenaufkommens von Februar auf März um etwa 4 Patienten pro Tag, bis April um etwa 10 Patienten pro Tag. Im Jahr 2020 stellt sich stattdessen ein deutlicher Abfall von Februar auf März um etwa 16 Patienten pro Tag, bis April sogar um etwa 21 Patienten pro Tag dar.

Im Fachbereich „Andere“ wurden im beobachteten Zeitraum des Jahrs 2019 insgesamt 78 Patienten behandelt, während es im Jahr 2020 etwa 166 Patienten waren. Von Februar bis April desselben Jahrs blieben die Zahlen sowohl in 2019 als auch in 2020 weitgehend konstant mit einer maximalen Schwankung von etwa 0,3 Patienten pro Tag.

Bezogen auf die 2 wichtigen Fachbereiche „Konservativ“ und „Chirurgie“ zeigt sich, dass im Jahr 2020 von Februar bis April ein zum Vorjahr überproportional deutlicher Rückgang der Patientenzahlen zu verzeichnen ist.

### Dringlichkeit

**Figure 3:**
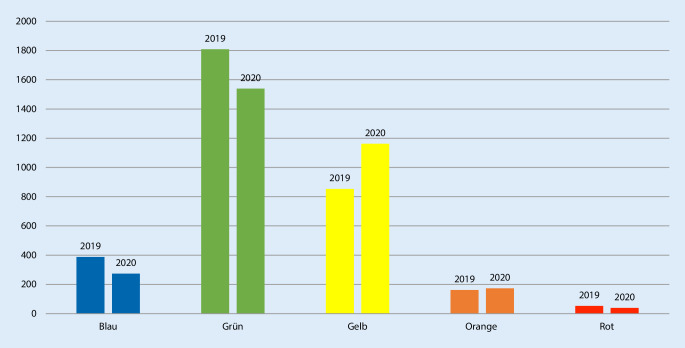

Vergleicht man das Patientenaufkommens der Jahre 2019 und 2020 miteinander, ist klar zu erkennen, dass die Dringlichkeitsstufe „gelb“ zugewinnt, wohingegen die Patientenzahlen für „blau“ und „grün“ zurückgingen (Abb. 3).

### Beschwerdebilder

In Tab. 3 ist die Verteilung der Notfälle anhand der 51 Beschwerdebilder gemäß MTS für den Beobachtungszeitraum 2019 und 2020 aufgeführt. Die häufigsten 3 Beschwerdebilder in den Monaten Februar bis April für die Jahre 2019 und 2020 waren demnach „Extremitätenprobleme“ (2350 vs. 2005 Patienten), „Unwohlsein bei Erwachsenen“ (1171 vs. 1745 Patienten) und „Abdominelle Schmerzen bei Erwachsenen“ (1019 vs. 915 Patienten).

**Table 3:** 

Beschwerdebild	2019			2020		
	Februar	März	April	Februar	März	April
Abdominelle Schmerzen bei Erwachsenen	287	360	372	381	276	258
Abdominelle Schmerzen bei Kindern	0	4	2	1	1	0
Abszesse und lokale Infektionen	60	85	91	67	80	45
Allergie	23	19	24	19	9	20
Angriff (Zustand nach)	24	16	28	34	14	11
Asthma	0	1	6	1	3	8
Atemproblem bei Erwachsenen	120	94	106	154	128	138
Atemproblem bei Kindern	0	1	0	0	0	0
Auffälliges Verhalten	7	2	5	10	1	9
Augenprobleme	27	32	24	92	80	87
Besorgte Eltern	0	0	0	0	1	0
Betrunkener Eindruck	6	2	9	9	7	7
Bisse und Stiche	16	39	33	28	17	36
Chemikalienkontakt	4	1	5	1	1	0
Diabetes	3	1	1	2	3	2
Durchfälle und Erbrechen	28	36	25	19	21	13
Extremitätenprobleme	668	819	863	841	683	481
Fremdkörper	6	14	14	10	5	4
Gastrointestinale Blutung	25	26	29	24	27	17
Generelle Indikatoren	287	319	363	141	152	127
Gesichtsprobleme	43	42	39	56	32	30
Halsschmerzen	18	12	16	16	22	16
Hautausschläge	6	10	13	6	3	3
Herzklopfen	19	19	23	58	43	27
Hodenschmerz	3	0	1	0	3	1
Körperstammverletzung	34	37	26	30	31	18
Kollaps	85	97	115	110	101	52
Kopfschmerz	114	141	159	164	129	102
Kopfverletzung	4	4	3	7	10	9
Krampfanfall	13	19	16	41	37	57
Misshandeltes Kind	0	0	0	0	1	0
Nackenschmerz	16	16	19	20	16	5
Ohrenprobleme	3	5	1	7	3	1
Psychiatrische Erkrankung	14	14	13	22	19	13
Rückenschmerz	2	0	0	3	1	2
Reanimation	143	169	168	184	100	78
Schreiendes Baby	0	1	0	0	0	1
Schwangerschaftsproblem	2	1	2	6	6	8
Schweres Trauma	5	2	15	8	11	10
Selbstverletzung	4	1	4	0	2	1
Sexualinfektion	4	5	4	0	1	0
Stürze	178	126	155	192	153	168
Thoraxschmerz	117	128	123	111	97	90
Überdosierung und Vergiftung	6	10	9	24	28	24
Unwohlsein bei Erwachsenen	365	387	419	684	568	493
Unwohlsein bei Kindern	0	1	1	2	2	0
Urologische Probleme	9	6	3	18	26	24
Vaginale Blutung	2	1	3	7	3	3
Verbrennungen und Verbrühungen	12	17	1	8	3	5
Wunden	125	190	166	168	135	153
Zahnprobleme	4	1	4	10	6	8
*Gesamtergebnis*	*2941*	*3333*	*3521*	*3796*	*3101*	*2665*

**Figure 4:**
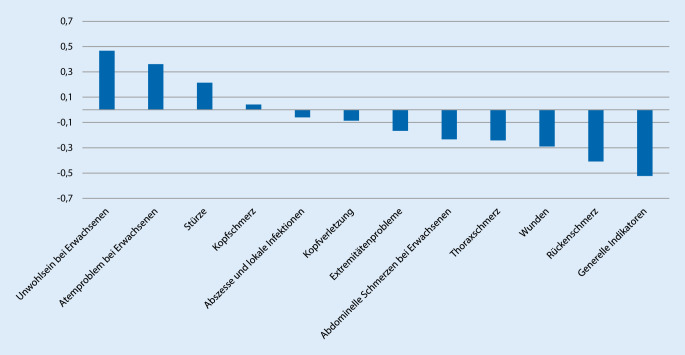

Um die Auswirkung der COVID-19-Pandemie auf die einzelnen Beschwerdebilder zu untersuchen, haben wir diese im März 2020, dem Monat mit dem höchsten Aufkommen von COVID-19-Patienten (Abb. 1), mit dem Vorjahr verglichen (Abb. 4). Hier zeigt sich eine deutliche Zunahme von „Unwohlsein bei Erwachsenen“ (+47 % zum Vorjahr), „Atemprobleme bei Erwachsenen“ (+36 % zum Vorjahr), während „Rückenschmerz“ (−41 % zum Vorjahr), „Wunden“ (−29 % zum Vorjahr) und „Thoraxschmerz“ (−24 % zum Vorjahr) im Vergleich zum Vorjahr deutlich abgenommen haben.

## Diskussion

Am Klinikum rechts der Isar stellen sich jedes Jahr mehr als 40.000 Patienten in der zentralen Notaufnahme vor. Vergleicht man die Patientenzahlen im untersuchten Zeitraum der Jahre 2019 und 2020 sind die Notfälle auf vergleichbarem Niveau mit einer leicht steigenden Tendenz in 2020 (Abb. 1). Es zeigt sich jedoch über alle Fachbereiche hinweg ein deutlicher Abfall an Notfallpatienten von Februar bis April 2020. Das tägliche Patientenaufkommen von 131 Patienten pro Tag im Februar fällt ab dem 10. März auf etwa 88 Patienten, also um etwa 32 %, für den restlichen Monat und hält bis einschließlich April an (Abb. 1). Zeitgleich mit den sinkenden Zahlen an Notfallpatienten stiegen die bestätigten COVID-19-Fälle im Münchener Raum und auch am Klinikum rechts der Isar. Ab dem 20. März werden über die ZNA täglich mindestens 4 bestätigte COVID-19-Fälle aufgenommen (Abb. 1).

Für den starken Einbruch an Notfallpatienten in der 2. Märzhälfte müssen mehrere Faktoren in Betracht gezogen werden. Hauptursächlich scheint jedoch der spürbare Beginn der COVID-19-Pandemie in Deutschland zu sein.

Betrachtet man die Fachbereiche getrennt voneinander, ist zu erkennen, dass die Anzahl der Notfallpatienten mit unfallchirurgischen Krankheitsbildern im Vergleich zu den konservativen Notfallpatienten mit Erkrankungen aus anderen Fachbereichen deutlich abgefallen ist (Abb. 2). Ursächlich hierfür könnte unter anderem die in Bayern ab dem 21.03.2020 verfügte Ausgangssperre sein [1]. Die Mobilität und sportlichen Aktivitäten sind hierdurch für die Bevölkerung deutlich eingeschränkt worden. Alle öffentlichen Spielplätze und Sportstätten, wie Fitnessstudios, Freizeitbäder, etc., wurden geschlossen und Mannschaftssportarten, wie Fußball, Basketball, etc., durften nicht mehr stattfinden. Ein Rückgang der Mobilität und sportlichen Aktivität könnte somit zu weniger Fällen mit unfallchirurgischen Krankheitsbildern führen.

Bestätigt wird dies durch eine deutliche Umverteilung der Beschwerdebilder, die im Rahmen der Triage erhoben werden. Wie in Abb. 4 zu erkennen haben im Vergleich zum Jahr 2019 im März 2020 beispielsweise Atemwegsprobleme deutlich zugenommen und demgegenüber Kopfverletzungen und Rückenschmerzen deutlich abgenommen. Derartige Schwankungen können ebenfalls saisonal bedingt sein, insbesondere durch die jährlich auftretende Influenza- und Respiratory-Sinzytial-Virus(RSV)-Welle. Diese fiel im Jahr 2020 im Vergleich zu den vergangenen 5 Jahren mit einer Dauer von 11 Wochen allerdings deutlich kürzer aus und endete am 22.03.2020 [13]. Ebenso sind die Todeszahlen im Jahr 2020 mit 518 influenzabedingten Todesfällen um etwa 54 % niedriger als im Vorjahr [13, 14].

Ein weiterer Grund für den Rückgang an Notfallpatienten könnte die sinkende Zahl an Selbsteinweisungen sein. Infolge der medialen Präsenz der COVID-19-Pandemie und des erhöhten Ansteckungsrisikos in Krankenhäusern wäre es denkbar, dass sich derartige Bedenken auf die Konsultationsfrequenz der Patienten auswirken. Die Bekanntgabe der ersten Todesfälle in Deutschland am 09. März könnte ebenfalls dazu beigetragen haben [12]. Denkbar wäre ebenso, dass Beschwerden hierdurch länger ausgehalten werden bzw. ganz auf einen Arztbesuch verzichtet wird.

Auch durch unterschiedliche Empfehlungen einzelner Fachgesellschaften, des RKI, der WHO, der Bundesländer und der EU-Staaten, herrscht unter der Bevölkerung Unsicherheit über den richtigen Umgang mit Schutzmaßnahmen im Rahmen der Pandemie. Durch fehlende soziale Kontakte könnten auch Faktoren wegfallen wie z. B. ein freundschaftlicher Rat, sich in ärztliche Behandlung zu begeben. Bei Fehlen dieser laienbasierten Unterstützung wird so der Gang ins Krankenhaus verzögert.

Auch die veränderte Verteilung der Dringlichkeit von „blau“ und „grün“ hin zu „gelb“, also zu einer zeitkritischeren Vorstellung, sowie die signifikante Zunahme der stationären Aufnahmen um 5 % im Jahr 2020 im Vergleich zum Vorjahr sprechen ebenfalls dafür, dass Patienten mit einer vermutlichen Bagatelle eher zu Hause bleiben und sich eher solche mit schwerwiegenderen Krankheitsbildern vorstellen. Es bleibt allerdings ungewiss, ob Patienten möglicherweise aufgrund dieser Bedenken so lange gewartet haben, bis eine ernstzunehmende Erkrankung erwachsen ist. Daten, die diese Vermutung erhärten könnten, wie z. B. die Verweildauer oder Dauer der ggf. nötigen intensivmedizinischen Behandlung, liegen leider nicht vor.

Ein sinnvoller Ansatz, um derartige Kollateralschäden, die möglicherweise durch einen verzögerten oder ausgesetzten Krankenhausbesuch entstehen, zu vermeiden, wäre, der Bevölkerung Ängste zu nehmen, die Kommunikation über Schutzausrüstung und Ansteckungsgefahr im Krankenhaus zu verbessern und auf die gute Infrastruktur des deutschen Gesundheitssystem zu verweisen. Denn trotz eines hohen Aufkommens an COVID-19-Patienten bestanden zu jedem Zeitpunkt ausreichende Kapazitäten, um alle Notfälle adäquat behandeln zu können.

### Limitationen

Im Rahmen der Auswertung des IT-Systems ERPath wurden keine weiterführenden Basisdaten zu den Patienten erhoben, daher kann auch keine Aussage zur tatsächlichen Krankheitsschwere der einzelnen Notfälle gemacht werden. Die Auswertung gibt auch keine Information über den weiteren Verlauf der Patienten. Daten zur Entlassart oder zum stationären Verlauf liegen nicht vor. Ebenso haben wir keinen Zugriff auf Daten aus dem ambulanten Sektor, die Aufschluss über die Auslastung der Hausarztpraxen geben könnten.

Warum die Patientenzahlen mit Beginn der Behandlung der ersten COVID-19-Fälle gesunken sind, kann nicht mit letzter Sicherheit geklärt werden.

Weiterführende Daten wie ICD-10-Hauptdiagnose oder stationäre Behandlungsdauer wären zur weiteren Bestimmung von möglichen Kollateralschäden von erheblicher Bedeutung.

## Fazit für die Praxis

- Das Patientenaufkommen in der Notaufnahme ist zur Zeit der COVID-19(Coronavirus-disease-2019)-Pandemie gesunken.
- Ursächlich könnten unseres Erachtens auch unbegründete Ängste in der Bevölkerung über eine mögliche Infektion im Krankenhaus sein.
- Bessere Kommunikation auf allen Kanälen könnte den Patienten die Sicherheit geben, sich im Notfall in ein Krankenhaus zu begeben.
- Bei bestehenden Krankenhauskapazitäten müssen Kollateralschäden der COVID-19-Pandemie vermieden werden.
